# Immunotherapy in small-cell lung cancer: from molecular promises to clinical challenges

**DOI:** 10.1186/s40425-019-0690-1

**Published:** 2019-08-05

**Authors:** A. Pavan, I. Attili, G. Pasello, V. Guarneri, P. F. Conte, L. Bonanno

**Affiliations:** 1 Medical Oncology 2, Istituto Oncologico Veneto IOV IRCCS, Via Gattamelata 64, 35100 Padova, Italia; 2 Department of Surgery, Oncology and Gastroenterology, Università degli Studi di Padova, Padova, Italia

**Keywords:** Small cell lung cancer, Immune checkpoint inhibitors, Immunotherapy, Tumor microenvironment, Combination therapy, Enhancer of zeste homolog 2

## Abstract

Management of small cell lung cancer (SCLC) has not changed over the last decades. In more recent years, alterations of DNA repair machinery and other molecular pathways have been identified in SCLC and preclinical data suggest that dysregulation of these pathways might offer new therapeutic opportunities.

While immune checkpoint inhibitors (ICIs) have had a major impact on the clinical outcome of several solid tumors, including non-small cell lung cancer, the potential role of ICIs is currently under investigation in SCLC and some promising data are available. However, several clinical and biological hurdles have to be overcome and predictive markers are still eagerly needed. Knowledge of molecular pathways specifically involved in SCLC growth and treatment resistance is essential for a more rational planning of new combinations including ICIs.

The present manuscript summarizes the current clinical evidence on immunotherapy in SCLC, describes the molecular bases underlying treatment resistance and discusses the potentialities and the rationale of different therapeutic combinations.

## Introduction and rationale

Small-cell lung cancer (SCLC) globally accounts for 13–15% of all lung malignancies. It is a highly aggressive neuroendocrine tumor, characterized by rapid growth and early tendency to widespread metastasis; stage IV disease represents over 70% of new diagnoses. Clinical onset is often associated with heavy symptomatic burden and rapid decline of overall health [1].

Chemo- and radiotherapy still represent the mainstay of treatment and an initial high responsiveness to such treatments is often observed [2, 3]. Recurrence, however, occurs very early in most cases, leading to a very dismal prognosis and a 5-year overall survival (OS) of 14.7–27.3% and 2.8% for early-stage (LD) and extended disease (ED), respectively [1, 4, 5].

Unfortunately, during the last three decades, life expectancy for SCLC patients has not improved, resulting in SCLC being defined as a recalcitrant cancer [6, 7].

In this disappointing scenario, there is a strong rationale for testing immune checkpoint inhibitors (ICIs), drugs that have changed the paradigm of treatment of non-small cell lung cancer (NSCLC) and other solid tumors in the latest years [8] (Table 1).

**Table 1:** Summary of immune-modulating drugs and their targets

Drug name	Drug target	Isotype	Source
Ipilimumab	CTLA-4	IgG1	Human
Nivolumab	PD-1	IgG4	Human
Pembrolizumab	PD-1	IgG4	Humanized
Atezolizumab	PD-L1	IgG1	Humanized
Durvalumab	PD-L1	IgG1	Human
Tremelimumab	CTLA-4	IgG2	Human
Avelumab	PD-L1	IgG1	Human
Utomilumab	CD137	IgG2	Human
INCAGN01876	GITR	IgG1	Humanized
INCAGN01949	CD134	IgG1	Human
Rovalpituzumab Tesirine	DLL3	IgG1	Humanized

*CTLA-4* Cytotoxic T-Lymphocyte Antigen 4, *PD-1* programmed cell death protein-1, *PD-L1* programmed death ligand-1, *GITR* Glucocorticoid-induced TNF-receptor-related protein

Epidemiological, biological and clinical features of SCLC suggest a potential efficacy of ICIs.

First of all, SCLC has a strong association with smoking status and exposure to cigarette smoking is a predictive factor for responsiveness to ICIs in NSCLC [9].

SCLC also harbors a high load of non-synonymous somatic mutations, so called Tumor Mutational Burden (TMB) [10]. This feature potentially results in the release of tumor neoantigens able to elicit an adaptive immune response against the tumor cells [11].

The capacity of SCLC to elicit immune response is also suggested by the presence of auto-immune paraneoplastic syndromes in about 20 to 40% of cases [12]. Tumor-enhanced immunity and neurologic paraneoplastic syndromes have been associated with better prognosis. In particular, in a recent study, median OS of SCLC patients without paraneoplastic syndromes was 9.5 months, versus 18 months for the patients with Lambert-Eaton syndrome [13, 14]. Even when a clinically overt paraneoplastic syndrome is not diagnosed, the mere presence of auto-antibodies is related to better outcome, reflecting the ability to elicit a humoral immune response [15].

On the other hand, there are specific clinical features of SCLC that may potentially limit the usefulness and the benefit of ICIs. First of all, SCLC is a rapidly progressive disease, requiring rapid tumor shrinkage with chemotherapy. Moreover, the majority of SCLC patients are symptomatic and require steroids and this is particularly true in case of superior vena cava syndrome and brain metastases [16, 17]. Chronic steroids are a known limitation for ICIs treatment [18].

For all these reasons, up to now, clinical data on the efficacy of monotherapy with ICIs in this disease are not so promising, in spite of a sound biological background. The antibodies used as immunotherapeutic agents belong to different IgG isotypes (Table 1). This may result in different activities since IgG1 are known to have stronger binding affinity to Fcϒ receptors compared to IgG2-3-4, thus able to mediate more effective antibody dependent cell-mediated cytotoxicity (ADCC). Despite pharmacological rationale, there are no demonstrated clinical differences among different isotypes; a reason can be found in the mechanism of action in relation to the immune target, since the action of anti-PD-1 antibodies can be independent from Fcϒ receptors [19].

Increasing evidence is available about molecular characterization and key pathways explaining specific features of immune-related microenvironment and key pathways responsible for the development of chemo-resistance.

In the manuscript we review molecular rationale for immunotherapy treatment, for synergism with chemotherapy and for other potential combination treatment including immunotherapy. We also summarize clinical evidence available and provide future potential perspectives.

## Molecular basis of chemo-resistance and synergism with immunotherapy

Cytotoxic drugs can induce an immunogenic cell death, leading to the generation of molecular signals that promote the uptake of dying cancer cells’ debris by antigen presenting cells (APC), and the cross-presentation of tumor antigens to T cells. Multiple molecular mechanisms induced by cancer cells, such as downregulation of major histocompatibility complex antigen expression, induction of an immunosuppressive milieu and negative regulation of cytotoxic T-cells via checkpoint inhibition, can inhibit this response. Combining ICIs with chemotherapy may disrupt these escape pathways and efficiently restore the anti-tumor activity of the immune system [20, 21]. In SCLC, however, the level of evidence in this field is still scarce and incomplete; a more comprehensive knowledge of the molecular basis of the mechanisms of resistance to chemotherapy and immunotherapy and of the expected activity of different chemo-immunotherapy combinations is needed.

**Fig. 1:**
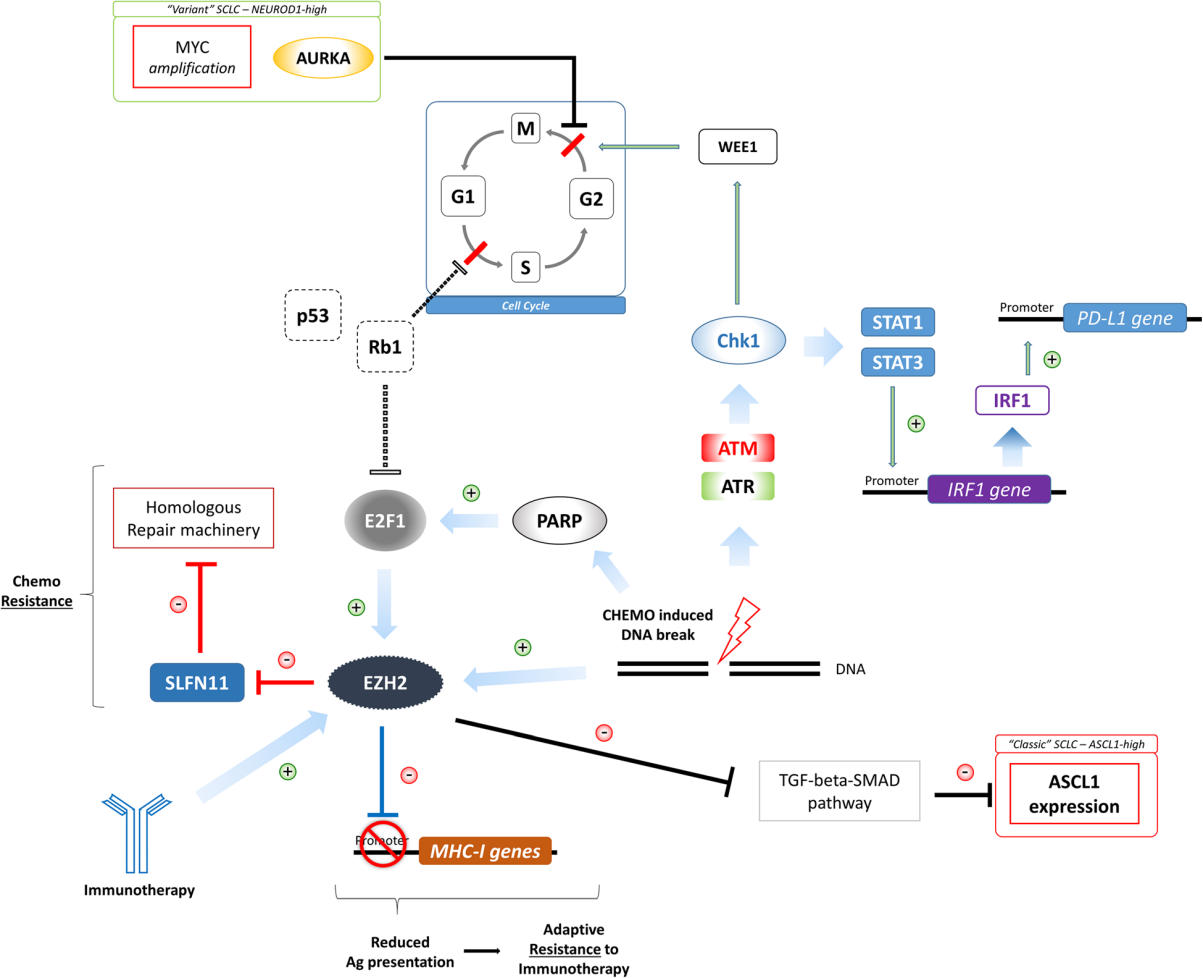
Molecular landscape of SCLC. SCLC cells are characterized by ubiquitous loss of TP53 and Rb1 (dotted lines), the main G1-S cellular cycle checkpoints. SCLC cells depend on G2-M cell cycle checkpoint, that may be influenced by Aurora kinase A over-expression, characterizing the Myc-driven “variant” subtype of SCLC) and by Chk1-WEE1 axis. Chk1 is activated by Ataxia telangiectasia Mutated (ATM)/Ataxia telangiectasia and Rad-3 related protein (ATR) pathway upon chemo-induced DNA double strand break. After its activation, Chk1 can induce G2 cell cycle arrest through the phosphorylation of WEE1. Activated Chk1 can also up-regulate PD-L1 expression through the activation of the Signal Transducer and Activator of Transcription 1–3 (STAT1–3) mediated regulation of Interferon regulatory factor 1 (IRF1). Signaling pathways involving Enhancer of zeste homolog 2 (EZH2), an epigenetic modifier inducible both by immunotherapy and cytotoxic agents, also seem crucial in SCLC. EZH2 activity is required for the acquisition of an immunosuppressive phenotype, down-regulating antigen presentation process (resistance to immune-therapy), and also for an enhanced chemo-resistance property, through the inhibition of Schlafen family member 11 (SLFN11), a negative regulator of homologous repair machinery (HRM)

SCLC cells are characterized by ubiquitous loss of tumor protein p53 (TP53) and Retinoblastoma 1 (Rb1), the main gatekeepers of G1-S transition [11]. This results into tumor cells arrest upon DNA damage at G2-M checkpoint with subsequent imbalance in expression and interaction of many DNA-damage response (DDR) proteins (Fig. 1) [22].

Checkpoint Kinase 1 (Chk1) is one of the main transducers of G2-M checkpoint activation. After its activation, Chk1 can induce G2 cell cycle arrest through the phosphorylation of WEE1 G2 checkpoint kinase (WEE1), among the others [23]. In SCLC cells, baseline Chk1 levels are higher than in controls, both in vitro and in human tissue samples [24], suggesting a crucial role of this protein for the control of tumor progression. Moreover, Chk1 is activated by Ataxia telangiectasia Mutated (ATM)/Ataxia telangiectasia and Rad-3 related protein (ATR) pathway upon chemo-induced DNA double strand breaks, thus resulting in unbalanced levels potentially leading to chemoresistance [25]. Intriguingly, Chk1 has been demonstrated to up-regulate Programmed death ligand 1 (PD-L1) expression, through the activation of the Signal Transducer and Activator of Transcription 1–3 (STAT1-3) mediated regulation of Interferon regulatory factor 1 (IRF1, [25–27]). This aspect can suggest a dynamic modulation of PD-L1 expression upon chemotherapy and a potentially greater benefit from a sequential instead of concomitant administration of immunotherapy.

In the latest years, the importance of Enhancer of zeste homolog 2 (EZH2)/Schlafen family member 11 (SLFN11) pathway has also been demonstrated in relationship with both chemotherapy and immunotherapy (Fig. 1).

The epigenetic modifier EZH2 is known to be induced by immunotherapy. In melanoma models, treatment with immune-modulating agents resulted in enhanced EZH2 activity [28]. Moreover, it has been demonstrated that immunotherapy can down-regulate the processes related to antigen presentation (Major Histocompatibility Complex-I, antigen processing, immunoproteasome subunits) and that EZH2 activity is required for the acquisition of this immunosuppressive phenotype [28]. On the other hand, SLFN11, whose activity is to silence the homologous repair machinery (HRM), is suppressed after chemotherapy in SCLC patient-derived xenograft (PDX), especially in chemo-resistant models [29]. EZH2 activity is required for SLFN11 suppression, thus suggesting its role also in chemoresistance. Consistently, the addition of an EZH2 inhibitor to platinum/etoposide chemotherapy in SCLC PDX models prevents the occurrence of resistance [29]. Interestingly, as a member of the HRM, poly ADP ribose polymerase (PARP) activity is also dysregulated in SCLC [30] and it is regulated by SLFN11 [31]. PARP inhibitors are active in SCLC models and clinical trials are ongoing [23, 32]. A phase II trial evaluating the addiction of veliparib, a PARP 1–2 inhibitor, to temozolomide in patients with recurrent SCLC showed no benefit in terms of PFS and OS; however, significantly higher objective response rate (ORR) was observed in patients receiving veliparib with temozolomide. Interestingly, patients with SLFN11-positive tumors obtained increase in PFS and OS if treated with the combination, while SLFN11-negative did not [33]. Another randomized phase II study, assessing the combination of veliparib with cisplatin and etoposide in first line treatment for ED-SCLC patients, failed to reach its primary endpoint of increasing PFS [34]. These different results may suggest the need of a predictive biomarker, in order to better exploit this class of drugs.

Aurora kinase A (AURKA) is a negative regulator of G2-M transition and is crucial in MYC amplified SCLC (around 20% of SCLC tumors) [35]: the inhibition of AURKA induces cell cycle arrest and strongly suppresses tumor growth in SCLC models (Fig. 1) [23, 36]. Moreover, AURKA may have a role in tumor cell growth and migration, through its interaction with the liver kinase B1 (LKB1). Zheng and colleagues have recently demonstrated that AURKA can directly phosphorylate LKB1 at position Ser299 in NSCLC models [37]. LKB1 phosphorylation prevents its interaction with AMP-activated protein kinase (AMPK), leading to a negative regulation of the LKB1/AMPK axis, which is normally responsible of tumor suppression [37, 38]. More in depth, LKB1 activity is crucial in regulating tumor cell metabolism, since it can modulate the intracellular levels of glutathione in response to oxidative stress [39]. The loss of LKB1 activity makes the tumor cell more sensitive to oxidative stress and consequently to stress-inducing treatments, such as chemotherapy and radiotherapy [40]. Skoulidis and colleagues recently demonstrated that KRAS-mutant lung adenocarcinomas harboring LKB1 co-mutations are associated with lower progression free survival (PFS) and OS to Protein death 1 (PD-1) blockade, thus suggesting a role of LKB1 in primary resistance to this class of drugs [41]. These data might suggest that AURKA-driven SCLCs are more sensitive to chemo-radiation treatments and resistant to ICIs.

## Role of tumor immune-microenvironment in SCLC

A body of evidence has been gathered over the years on the role of the tumor immune microenvironment (TME), i.e. the milieu of lymphocytes, monocytes and other immune cells intertwined with cancer cells, in neoplastic initiation and progression. The composition of the TME differs across time and stages even in cancers with same histology and it is one of the determinants of tumor characteristics and outcome of NSCLC patients [42].

An early study focusing on the interaction between SCLC cells and their TME showed how SCLC tumor cell lines were able to inhibit activated CD4+ T-cells [43]. The inhibitory activity did not require a direct cell-to-cell contact, but was mediated by cytokine secretion by tumor cells (IL-15 in particular) that caused a de novo functional differentiation of lymphocytes towards a T-regulatory immunophenotype (FOXP3+ CD4+ T-cells). Another study has analyzed FOXP3+ infiltrate in archival biopsies from patients with SCLC and the ratio of FOXP3+ turned out to be an independent indicator of poor prognosis in these patients [43].

The histological assessment of SCLC TME was the focus of another study that evaluated the prognostic role of CD45 (a pan-inflammatory cell marker) positive immune cells [44]. The extent of CD45+ infiltrate was predictive of a longer OS independently from clinical parameters such as stage and performance status [45].

Increasing evidence has indicated that TME is able to modulate the PD-1/PD-L1 axis, promoting the innate tendency of cancer cells to escape immune-surveillance [46]. Data on distribution of PD-L1 expression in SCLC across stages are very limited; in patients with advanced disease the level of PD-L1 expression seems to be lower than in earlier stages [47, 48] and also than in NSCLC [49].

A retrospective study conducted in ED-SCLC and LD-SCLC patients treated with a multimodal approach, including surgery for early stage, showed an association between CD8+ tumor infiltrating lymphocytes (TILs) and PD-L1 expression on tumor cells, whereas FOXP3+ infiltrate showed a positive correlation with PD-L1 positive tumor-infiltrating T cells [48]. Furthermore, a stronger infiltration of FOXP3+ TILs characterized early stage disease and was associated with a better prognosis in LD-SCLC patients, shedding a new light upon the controversial role of the T regulatory subset of TILs even in this malignancy [48, 50, 51].

## Immune checkpoint inhibitors in SCLC: clinical perspectives

### First line

Only few data are available on ICIs as monotherapy in first line setting, because of the potential risks of not administering chemotherapy in such a rapidly progressive disease. For this reason, taking into account the potential synergism [20, 21], most trials have explored the combined approach of chemotherapy and immunotherapy.

In a randomized phase II study, patients with untreated ED-SCLC were randomized to receive chemotherapy (carboplatin plus paclitaxel) with either placebo (control arm) or ipilimumab in two alternative regimens, concurrent with chemotherapy (concurrent arm) or sequential (phased arm). In this trial the addition of ipilimumab conferred only a minimal increase in immune-related PFS for patients who received phased-ipilimumab compared to placebo, but not for patients receiving concurrent treatment [52].

Subsequently, a randomized phase III study combining ipilimumab with platinum plus etoposide failed to demonstrate a benefit in PFS or OS [53].

Despite these first disappointing results, the path of combination strategy was further pursued. IMpower133, a phase III double blind randomized trial, evaluated the efficacy and safety of atezolizumab added to carboplatin and etoposide as first-line treatment for patients with ED-SCLC. A total of 403 patients were randomized to receive atezolizumab plus chemotherapy followed by atezolizumab maintenance treatment or chemotherapy plus placebo [54]. The study met both its co-primary endpoints, showing statistically significant improved OS and PFS. The magnitude of the benefit, however, was not impressive (2 months in median OS and 0,9 month in median PFS), with no sign of plateauing of survival curve, as previously seen for NSCLC [55, 56]. Nevertheless, the latest National Comprehensive Cancer Network (NCCN) guidelines included this chemo-immunotherapy regimen as a first line option for ED-SCLC patients [57] and the combination has been recently approved by FDA.

Several clinical trials are currently exploring, in first line treatment, the combination of PD-1/PD-L1 inhibitors with chemotherapy and other ICIs, as summarized in Table 2.

**Table 2:** Ongoing clinical trials with immune checkpoint inhibitors in first line setting for SCLC

Clinical trial ID	Phase	Setting	Regimen	Endpoint
NCT03382561	II	ED-SCLC	CE vs CE plus nivolumab	PFS
NCT02580994 (REACTION)	II	ED-SCLC	CE vs CE plus pembrolizumab	PFS
NCT02934503	II	ED-SCLC	CE plus pembrolizumab +/− RT	PD-L1 changes
NCT03066778 (KEYNOTE-604)	III	ED-SCLC	CE plus pembrolizumab/placebo	PFS OS
NCT02763579	III	ED-SCLC	CE plus atezolizumab/placebo	PFS OS
NCT03043872	III	ED-SCLC	CE vs CE plus durvalumab +/−tremelimumab	PFS OS
NCT02402920	I	LD-SCLC	CE-RT plus pembrolizumab	MTD

*ED-SCLC* Extensive-stage Disease Small cell lung cancer, *CE* Cisplatin/Carboplatin plus Etoposide, *PFS* Progression Free Survival, *DLTs* Dose-limiting Toxicities, *OS* Overall Survival, *AEs* Adverse Events, *ORR* Overall Response Rate, *LD-SCLC* Limited-stage Disease Small cell lung cancer, *RT* Radiotherapy, *MTD* Maximum Tolerated Dose

Another promising approach is represented by the association of radiotherapy and immunotherapy. Similarly to chemotherapy, radiation therapy can induce an immunogenic cell death [21, 58]. Clinical trials are also evaluating concurrent administration of radiotherapy and chemo-immunotherapy regimens containing pembrolizumab (NCT02934503, NCT02402920, https://www.clinicaltrials.gov).

The association of the anti-PD-L1 durvalumab with the anti-CTLA4 tremelimumab is also under investigation (NCT02658214, NCT03043872, https://www.clinicaltrials.gov). The rationale behind this combination is to exploit the different mechanisms of action: inhibiting CTLA-4 leads to differentiation of naïve T cells, which will later be able to infiltrate tumor tissues with no restraint on their anti-tumor activity mediated by PD-1/PD-L1 inhibition [59].

### Maintenance

While it is hard to replace first line chemotherapy, the rapid decline of performance status and the worsening of symptoms at disease progression might prevent many patients to be eligible for immunotherapy as salvage treatment. Moreover, chemotherapy may enhance the susceptibility of the tumor to immunotherapy: all these features represent the rationale of administrating ICIs as a maintenance or consolidation treatment. A phase II single arm trial assessed the efficacy of maintenance pembrolizumab in 45 ED-SCLC patients, after response or stable disease following platinum/etoposide chemotherapy [60]. Maintenance started within 8 weeks from the last cycle of chemotherapy and continued for a total of 2 years. The primary endpoint was the improvement of median PFS to 3 months (50% increase over 2 months of the historical controls). The endpoint was not met, with a median PFS of 1.4 months (95%CI: 1.3–2.8 months); however, a subset of patients with any PD-L1 expression on cells confined in the stromal interface could gain a long lasting benefit from maintenance (6.5 months, 95%CI: 1.1–12.8 months) [60].

The same setting of treatment was evaluated in CheckMate 451 study [61]. In this phase III trial, patients with ED-SCLC, who achieved disease control after first-line platinum-based chemotherapy, were randomized to receive nivolumab alone (240 mg every 2 weeks), nivolumab (1 mg/kg every 3 weeks) with ipilimumab (3 mg/kg every 3 weeks) up to 4 cycles, followed by nivolumab (flat 240 mg every 2 weeks), or placebo until disease progression or unacceptable toxicity, for a maximum of 2 years. Primary endpoint was OS improvement for patients treated with ICI combination versus placebo. This endpoint was not met, with a disappointing median OS for the ipilimumab and nivolumab group of 9,2 months (95% CI: 8,2–10,2 months) versus 9,6 months (95% CI: 8,2–11 months) of the placebo group. This trial showed many critical issues, the first one being the fact that almost 60% of the patients received maintenance after 5 weeks or more from the last dose of first line chemotherapy [61]. Furthermore, unlike phase III NSCLC trials [62], here the dosage of the ipilimumab was 3 mg/kg, this fact being responsible of a median number of 2 doses administered to patients of the combination arm. Further analyses are in progress, in order to identify possible subgroups of patients who may benefit from ICI doublet as maintenance strategy.

A summary of the ongoing clinical trials in maintenance setting is reported in Table 3.

**Table 3:** Ongoing clinical trials in maintenance or consolidation setting after first line treatment for SCLC

Clinical trial ID	Phase	Setting	Regimen	Endpoint
NCT02046733 (STIMULI)	II	LD-SCLC, after C-RT	Nivolumab plus ipilimumab	OS PFS
NCT03043599	I/II	LD-SCLC, after Cx	Nivolumab plus ipilimumab plus RT	RP2D PFS

*LD-SCLC* Limited-stage Disease Small cell lung cancer, *C-RT* chemo-radiation, *OS* Overall Survival, *PFS* Progression Free Survival, *Cx* Chemotherapy, *RT* Radiotherapy, *RP2D* Recommended Phase 2 Dose, *ED-SCLC* Extensive-stage Disease Small cell lung cancer

### Beyond first line

Recurrence after first line treatment is almost inevitable and few effective options at the time of progression are available. Response rate to standard second line chemotherapy is 24.3%, with a median duration of response (DOR) of around 14 weeks, at the cost of grade 3 and 4 toxicities [63]. CheckMate 032 was the first trial to evaluate immunotherapy for SCLC patients who had failed a first line platinum-based chemotherapy [49]. In this phase I/II open label trial, 216 patients were randomized to receive nivolumab alone (3 mg/kg bodyweight every 2 weeks), or different combination of nivolumab/ipilimumab (1 mg/kg plus 1 mg/kg, 1 mg/kg plus 3 mg/kg, or 3 mg/kg plus 1 mg/kg). Primary endpoint was the objective response (OR). An OR was achieved in 10, 23 and 19% of patients treated with nivolumab alone, nivolumab 1 mg/kg plus ipilimumab 3 mg/kg and nivolumab 3 mg/kg plus Ipilimumab 1 mg/kg respectively. Response rates were not related to PD-L1 expression on tumor cells, platinum-resistance or number of previous treatments. DOR was remarkable in every cohort, with nivolumab alone group still not reaching its median value at the time of the analysis. Safety profile was manageable, with fewer treatment-related toxic effects compared with previous trials of topotecan or amrubicin [64]. On the basis of the trial results FDA recently approved nivolumab for the treatment of SCLC in third line setting.

On the other hand, CheckMate 331 (NCT02481830), an open-label phase III trial, compared nivolumab versus standard of care chemotherapy as second line treatment for patients with SCLC progressing after first line platinum-based chemotherapy. The primary endpoint was OS and was not met. However, the authors highlighted that OS curves separate after 12 months, thus suggesting an important role for a subpopulation of patients who may derive prolonged clinical benefit, even in the presence of platinum-resistance [65].

In line with these promising results, Keynote 028, a phase Ib trial tested the activity and safety of pembrolizumab (given at 10 mg/kg every 2 weeks) in 24 extensive-stage SCLC patients selected for PD-L1 expression (TPS ≥ 1%), who had failed at least one line of standard therapy [66]. Overall response rate (ORR) and DOR were 33.3% and 19.4 months respectively; only eight patients experienced grade ≥ 3 immune-related adverse events (irAEs).

Results from the SCLC arm of Keynote 158, a phase II trial of pembrolizumab (flat dose of 200 mg every 3 weeks) in 107 pre-treated advanced SCLC patients [67], showed an ORR of 3.7% and a DOR of over 15 months (median DOR still not reached). Patients with a positive PD-L1 combined score achieved a better response (ORR: 35%), with an astonishing median OS of 14.6 months [68]. Results from a pooled analysis of these two clinical trials, Keynote 028 and 158, were recently presented. The ORR was 19.3% and median DOR was not reached. Two patients had a complete response and 14 had a partial response; 14 of 16 responders were PD-L1-positive. Median PFS and OS were 2 and 7.7 months respectively [69]. Based on these data FDA has granted the accelerated approval to pembrolizumab for patients with advanced SCLC with disease progression on or after platinum-based chemotherapy and at least one other prior line of therapy.

Anti-PD-L1 agents started being tested in similar treatment setting. Phase Ia study of atezolizumab in ED-SCLC patients relapsed after platinum-based chemotherapy with etoposide, showed a good safety profile of the drug, with encouraging results also in terms of efficacy and outcome, with confirmed ORR of 6%, median PFS of 1.5 months and median OS of 5.9 months [70]. A subsequent phase II trial, however, investigating the role of atezolizumab as second line treatment option, did not meet its primary endpoint of increased ORR with the anti-PD-L1 agent versus standard of care (i.e. topotecan or re-induction with carboplatin and etoposide, following investigator choice) [71]. PFS data were also quite disappointing: median PFS was 1.4 months in the atezolizumab group and 4.2 months in the chemotherapy one, with an unfavorable risk of progression (Hazard Ratio of 2,26, *p* = 0,004) for the experimental arm.

The first results of another anti-PD-L1 agent, durvalumab (10 mg/kg every 2 weeks), are also available. The study was performed in a PD-L1-unselected population. Primary endpoint was safety: treatment was well tolerated and all irAEs were grade 1 or 2. Secondary endpoints were also of interest with an ORR of 9.5%, a median PFS 1.5 months and a median OS 4.8 months [72]. Durvalumab showed a tolerable safety profile and a promising activity also when combined with tremelimumab, an anti-CTLA-4 agent. Initial data from a phase I dose-finding trial on heavily pre-treated ED-SCLC patients showed a 23% grade 3–4 irAEs, with a confirmed ORR of 13.3% and a median DOR of over 18 months [73].

Combination strategies have also been investigated after the failure of platinum-etoposide treatment. Positive findings about chemotherapy plus anti-PD-1 drug are coming from a phase II study that investigated the efficacy of this combination in a small group of platinum refractory ED-SCLC patients. Paclitaxel (175 mg/m2) was administered every 3 weeks up to 6 cycles and flat dose pembrolizumab (200 mg every 3 weeks) was added from the second cycle and continued until disease progression or unacceptable toxicity. ORR was 23.1%, with a disease control rate (DCR) of over 80% and median OS of 9.2 months. Toxicity was acceptable and main grade 3–4 events, such as febrile neutropenia, were related to chemotherapy [74].

A large number of trials are ongoing for this setting of treatment. ICIs are administered as single agent in single arm trial, as a single agent compared to standard treatment, or in combination either with other ICIs, or with chemotherapy, radiotherapy, or with other drugs (Table 4).

**Table 4:** Ongoing clinical trials in further lines of treatment for SCLC

Clinical trial ID	Phase	Setting	Regimen	Endpoint
NCT02963090	II	2nd line	Pembrolizumab vs topotecan	PFS
NCT03059667	II	2nd line	Atezolizumab vs standard chemotherapy	RR (non-comparative)
NCT02331251 (PembroPlus)	Ib/II	≥ 2nd line (solid tumors)	Pembrolizumab plus irinotecan	RP2D
NCT03253068	II	2nd line	Pembrolizumab plus Amrubicin	ORR
NCT02551432	II	≥ 2nd line	Pembrolizumab plus Paclitaxel	RR
NCT03262454	II	2nd line	Atezolizumab + RT	RR
NCT03026166	I/II	≥ 2nd line	Rova-T plus Nivolumab +/− ipilimumab	DLT
NCT03083691 (BIOLUMA)	II	2nd line	Nivolumab plus ipilimumab	ORR
NCT02701400	II	2nd -3rd line	Tremelimumab plus Durvalumab +/− RT	PFS ORR
NCT02554812	Ib/II	≥ 2nd line	Avelumab plus utomilumab	DLT OR

*OS* Overall Survival, *PFS* Progression Free Survival, *RR* Response Rate, *RP2D* Recommended phase 2 dose, *Rova-T* Rovalpituzumab Tesirine, *ORR* Overall Response Rate, *MTD* Maximum Tolerated Dose, *DLT* Dose limiting toxicities

## New partners for ICIs

In order to increase the therapeutic role of ICIs in SCLC, biological rationale supports the potentiality of combining ICIs with a number of non-chemotherapy agents with the aim to obtain synergism and subsequently improve both the percentage of patients who benefit from immunotherapy and the duration of clinical benefit (Table 5).

**Table 5:** Ongoing clinical trials of immune-checkpoint inhibitors combined with non-cytotoxic agents

Clinical trial ID	Phase	Setting	Regimen	Endpoint
NCT03041311	II	1st line	CE plus atezolizumab +/− Trilaciclib	OS AEs
NCT02922764	I	2nd line (solid tumors)	RGX-104 +/− Nivolumab	MTD ORR PFS
NCT02712905	I/II	≥ 2nd line (solid tumors)	INCB059872 plus Nivolumab (part 3)	AEs
NCT03126110	I/II	≥ 2nd line	INCAGN01876 plus nivolumab/ipilimumab/nivo+ipi	AEs ORR
NCT03241173	I/II	≥ 2nd line	INCAGN01949 plus nivolumab/ipilimumab/nivo+ipi	AEs ORR
NCT03026166	I/II	≥ 2nd line	Rova-T plus Nivolumab +/− ipilimumab	DLT
NCT03085849	I	2nd line	SGI-110 followed by Durvalumab plus Tremelimumab	MTD
NCT02734004 (MEDIOLA)	I/II	2nd line	Durvalumab plus olaparib	DCR ORR AEs
NCT02554812	Ib/II	≥ 2nd line	Avelumab plus utomilumab	DLT OR

*PFS* Progression Free Survival, *ORR* Overall Response Rate, *RGX-104* LXR inhibitor, *MTD* Maximum Tolerated Dose, *INCB059872* LSD1 inhibitor, *AEs* Adverse Events, *Rova-T* Rovalpituzumab Tesirine, *DLT* Dose limiting toxicities, *INCAGN01876* anti-GITR antibody, *INCAGN01949* anti-OX40 antibody, *SGI-110* DNMT inhibitor, *Trilaciclib* CDK4/6 inhibitor, *Utomilumab* anti-CD137 antibody

A first strategy concerns the idea that immune-tolerance mechanisms are redundant and that inhibiting more immune-suppressive targets may enhance anti-tumor activity. This is the most explored strategy and studies with the combination of nivolumab and ipilimumab have been described before.

**Fig. 2:**
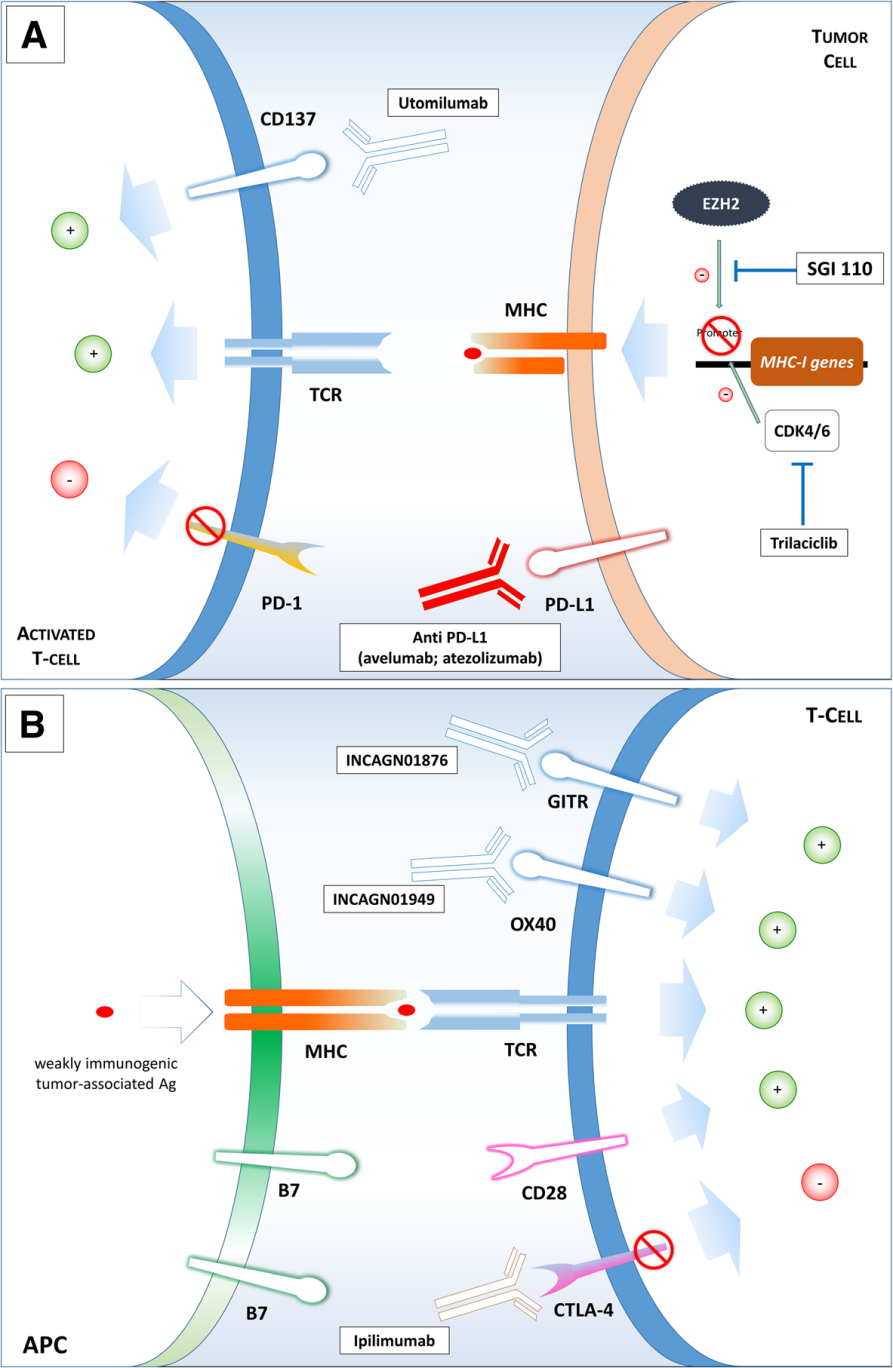
New combination strategies. Mechanisms of action of drugs that are being studied for new combination strategies in small-cell lung cancer. Panel **a**: utomilumab triggers CD137, a co-stimulatory receptor expressed on activated immune cells and it is studied in combination with avelumab; trilaciclib is a CDK4/6 inhibitor and it is studied with platinum/etoposide and atezolizumab; SGI110 contrasts the role of EZH2, by interfering with DNA methylation and it is under evaluation in combination with durvalumab. Panel **b**: another promising strategy is to associate immune checkpoint inhibitor, such as Ipilimumab, to immune stimulatory agents. INCAGN01876 is a monoclonal antibody that activates Glucocorticoid-induced TNF-receptor-related protein (GITR), a T cell co-stimulatory receptor involved in the immunological synapsis able to enhance T cell responsiveness to weakly immunogenic tumor-associated antigens. INCAGN01949, another antibody that targets and stimulates OX40, a T cell co-stimulatory receptor that potentiates TCR signalling

On the other side, new drugs are under evaluation with the aim of actively promoting immune-response in combination with anti-PD1/PD-L1 antibody. For example, Utomilumab is a fully human IgG2 agonist monoclonal antibody targeting CD137, a co-stimulatory receptor expressed on activated immune cells (effector and regulatory T cells, NK cells and dendritic cells), causing an enhanced cytotoxic T-cell and NK-cell activity [75] and triggering antitumor response [76] (Fig. 2). In this case, rationale for synergism is strong: anti-PD-1/PD-L1 disrupts the PD1/PD-L1 interaction, thus avoiding tumor-induced anergy of tissue infiltrating lymphocytes, while utomilumab can boost the anti-tumor activity of different effector white blood cells.

Other drugs act as co-stimulatory agents for T cell receptor (TCR) signaling: INCAGN01876, able to bind Glucocorticoid-induced TNF-receptor-related protein (GITR) (NCT03126110, https://www.clinicaltrials.gov), a T cell costimulatory receptor involved in the immunological synapsis during CD4+ and CD8+ T-cell priming, and INCAGN01949 (NCT03241173, https://www.clinicaltrials.gov), a fully human IgG1 monoclonal antibody that targets and stimulates OX40 (CD134), another T cell co-stimulatory receptor that potentiates TCR signaling in different processes (T-cell priming, effector cell differentiation and memory T cell recall responses).

A different strategy concerns the exploiting of other mechanisms not directly interacting with immune cells, but anyhow able to affect immune response. This is also the idea at the basis of combining chemotherapy and ICIs. Recently, the role of CDK4/6 (Cyclin-dependent kinase 4/6) is emerging in this context. This class of molecules, through the interaction with DNA-methyltransferase 1 (DNMT1), is responsible of increasing the immune-evasive T-cell phenotype [77]. The combination of platinum/etoposide and atezolizumab with the new molecule Trilaciclib, a CDK4/6 inhibitor, is currently in phase 2 clinical trial (NCT03041311, https://www.clinicaltrials.gov) (Fig. 2). Another interesting trial evaluates the combination of nivolumab and RGX-104, a small agonist ligand of liver-X receptors (LXRs) (NCT02922764 https://www.clinicaltrials.gov). LXRs belong to the nuclear receptor family and are able to regulate cellular proliferation; previous studies have shown that LXR-ligands have anti-cancer activities in a variety of cancer cell lines [78], they can induce immunogenic cell death [79] and modulate inflammatory response. In particular RGX-104 is able to deplete myeloid derived suppressor cells (MDSCs), stimulates dendritic cells and activates cytotoxic lymphocytes. The immunologic and anti-tumor activity of this drug has been demonstrated in patients with advanced refractory solid tumors and now a dose-escalation phase with nivolumab has started [80].

As mentioned before, EZH2 activity is crucial for SCLC, as it is involved in tumor sensitivity both to chemotherapy and to immunotherapy. EZH2 works mainly through histone modification and DNA-methylation. SGI-110 is a DNA methyltransferase inhibitor composed of a dinucleotide of decitabine and deoxyguanosine, that is currently being tested with durvalumab and tremelimumab in patients with ED-SCLC progressive after a platinum-based first-line chemotherapy (NCT03085849 https://www.clinicaltrials.gov) (Fig. 2). This kind of approach may be particularly promising since EZH2 is also involved in chemo-resistance mechanisms, as described before, and it is a pathway specifically involved in SCLC.

## Safety of combination treatments

Immune-related toxicity represents a major concern in SCLC. Autoimmune disorders are indeed frequent in SCLC patients, who may develop autoimmune diseases as paraneoplastic syndromes [12]. In this scenario, the relation between immune-related toxicities and treatment response might be intriguing, although data are scanty since patients with autoimmune disorders were excluded from clinical trials. To address this issue, retrospective series mainly involving NSCLC and melanoma patients have been described [81, 82]. Patients with active or inactive autoimmune disease have been treated with anti-PD1/anti-PD-L1 or anti-CTLA4. An autoimmune disease flare, mostly low grade and rarely requiring systemic corticosteroids, has been reported by about 20% of patients and this did not affect treatment outcome [81, 82]. Overall, the risk of immune-related adverse events was higher among patients with pre-existing autoimmune conditions, but the toxicity had no impact on survival [82]. No cases of paraneoplastic auto-immune syndromes were included in these series [81, 82].

In the CheckMate 032 trial with combined nivolumab and ipilimumab, the most frequent adverse events were increased lipases and diarrhea [49]. A peculiar, although rare, toxicity, was limbic encephalitis and aseptic meningitis across all treatment arms, while rash and hypothyroidism, mainly low-grade, were more frequently reported in the nivolumab-ipilimumab combination arms [49]. Rash and hypothyroidism were also the most common irAEs observed in the IMpower133 trial in the chemotherapy plus atezolizumab arm [54].

Pulmonary toxicity from the association of ICIs with chest radiotherapy may also be an issue. However, in the PACIFIC study, investigating durvalumab after chemo-radiation in stage III NSCLC, there were no differences in the incidence of grade 3 and 4 pneumonitis between the durvalumab and the placebo group [83].

In our experience, treatment with second-line nivolumab in a SCLC patient who had previously received thoracic radiotherapy for limited disease showed an exceptional clinical and radiological response. In the same patient, treatment was interrupted after 6 doses due to the occurrence of pneumonitis. The patient experienced single-site progression and received radiotherapy on a peri-pancreatic lymph-node. After the radiotherapy, he experienced further response on liver lesions and a relapse of immune-related pneumonitis, seven months after the completion of nivolumab treatment [84]. This experience shows how complicated are the effects of immune-modulation induced by cancer treatments and that the administration of radiotherapy also after ICIs and at distant-site may elicit immune-related adverse events.

## Predictive biomarkers of response to immune-checkpoint inhibitors in SCLC

Several trials have included correlative studies in order to find potential predictive markers of response.

In a trial combining ipilimumab 10 mg/kg with carboplatin and etoposide, the relation between baseline positivity of autoantibodies and clinical outcomes was evaluated. Patients with any positive autoimmune antibody (anti-SOX2, anti-Hu, anti-Yo, anti-VGCCA, anti-VGPCA, anti-nuclear, anti-neutrophil cytoplasmic antibodies) showed a trend for a prolonged survival (18.5 versus 17 months, *p* = 0.144), a significantly longer median progression free survival (8.8 versus 7.3 months, *p* = 0.036) and a trend for a higher response rate (*p* = 0.066) [85].

Differently from NSCLC trials, tumor PD-L1 expression in the Checkmate 032 was not predictive of ICIs efficacy in patients with SCLC [49]. Given this finding, samples were further analyzed: whole exome sequencing was performed and the tumor mutation burden was defined as the total number of non-synonymous somatic mutations [86]. Patients harboring higher tumor mutational load (defined as higher than the upper tertile of the mutation distribution of the study population) experienced an enhanced efficacy from the treatment, especially when the combination was administered.

Due to the limited availability of adequate tissue, there is an increased interest to use blood-based tests through cell free tumor DNA profiling. A blood-based surrogate of tissue-based tumor mutation burden evaluation has been shown to be a potential predictive tool for advanced NSCLC patients treated with atezolizumab [87]. Differently from NSCLC patients, patients with SCLC treated with atezolizumab plus carboplatin and etoposide showed a benefit in terms of OS and PFS, regardless of blood-based tumor mutational burden [54].

A retrospective study has evaluated tissue mutational burden (defined as total number of nonsynonymous mutations) of 120 patients with SCLC of all stages and the association with PD-L1 expression both on tumor and on immune cells [88]. Tissue mutational burden had no particular relationship with tumor expression of PD-L1, whereas there was a positive correlation with PD-L1 expression on immune infiltrate (*p* = 0.04). Gadgeel et al. have studied PD-L1 expression of cells confined in the tumor stroma of patients receiving pembrolizumab as a maintenance treatment after first line chemotherapy [60]. The stromal interface was considered PD-L1 positive if PD-L1 membrane-stained cells surrounding the tumor nests were identified at low power magnification. Patients with PD-L1 expression at the stromal interface had longer median PFS and median OS than patients with no expression (6.5 versus 1.3 months and 12.8 versus 7.6 months respectively). Exploratory analysis performed in the SCLC cohort of Keynote 158 has shown the potential of the PD-L1 combined score, i.e. the ratio of PD-L1 positive cells, including tumor cells, lymphocytes and macrophages, to the total number of tumor cells [67]. This PD-L1 score was able to define a subset of pre-treated ED-SCLC patients who achieved a better ORR (35.7% versus 6%), 1-year PFS (28.5% versus 8.2%) and 1-year OS (53.1% versus 30.7%) while on pembrolizumab.

## Conclusions

The systemic treatment of SCLC represents a major challenge for medical oncologists and immunotherapy has a great appeal and solid biological rationale.

Initial clinical experiences confirm the potentialities of ICIs for this aggressive disease and indicate the need for reliable predictive biomarkers. Preliminary data suggest that predictive biomarkers of ICIs efficacy might be disease-specific and that findings validated in NSCLC cannot be translated in SCLC. In fact, a different evaluation score for PD-L1 expression has been suggested.

Responsiveness to immunotherapy is related to clinical disease course and to the host, but also to biological features of the disease. The study of molecular mechanisms at the basis of chemo-resistance and aggressiveness of the disease may help in understanding also the immune-resistance mechanisms and in individuating new combinations treatment strategies with the aim of improving clinical benefit of immunotherapy.

In addition to combining ICIs with chemotherapy and immunotherapy, new therapeutic approaches, specifically addressing molecular pathways involved in SCLC growth and chemo-resistance, need to be explored in order to contribute to improve the outcome of SCLC patients, commonly recognized as an unmet clinical need.

## Data Availability

Not applicable.
